# Design and Reproducibility of a Mini-Survey to Evaluate the Quality of Food Intake (Mini-ECCA) in a Mexican Population

**DOI:** 10.3390/nu10040524

**Published:** 2018-04-23

**Authors:** María Fernanda Bernal-Orozco, Nayeli Badillo-Camacho, Gabriela Macedo-Ojeda, Montserrat González-Gómez, Jaime Fernando Orozco-Gutiérrez, Ruth Jackelyne Prado-Arriaga, Fabiola Márquez-Sandoval, Martha Betzaida Altamirano-Martínez, Barbara Vizmanos

**Affiliations:** 1 Bachelor of Nutrition, Centro Universitario de Ciencias de la Salud (CUCS), Universidad de Guadalajara (UdeG), Sierra Mojada 950, Building “N”, Colonia Independencia, Guadalajara ZC 44340, Mexico; fera_18@yahoo.com.mx (M.F.B.-O.); nuta.nayeli@yahoo.com.mx (N.B.-C.); gabriela.macedo@cucs.udg.mx (G.M.-O.); mgonzalez_investmed@hotmail.com (M.G.-G.); jfog97@live.com.mx (J.F.O.-G.); jacky.pa.51@gmail.com (R.J.P.-A.); fabiola_msandoval@yahoo.com.mx (F.M.-S.); martha_alt@yahoo.com.mx (M.B.A.-M.); 2 Doctorate in Traslational Nutritional Sciences, CUCS, UdeG, Juan Díaz Covarrubias and Salvador Quevedo y Zubieta, Building “C”, Colonia Independencia, Guadalajara ZC 44340, Mexico; 3 Doctorate in Public Health Sciences, CUCS, UdeG, Sierra Mojada 950, Building “N”, Colonia Independencia, Guadalajara ZC 44340, Mexico

**Keywords:** reproducibility, quality of food intake, food survey

## Abstract

Evaluating food intake quality may contribute to the development of nutrition programs. In Mexico, there are no screening tools that can be administered quickly for the evaluation of this variable. The aim was to determine the reproducibility of a mini-survey designed to evaluate the quality of food intake (Mini-ECCA) in a Mexican population. Mini-ECCA consists of 12 questions that are based on Mexican and international recommendations for food and non-alcoholic beverage intake, with the support of photographs for food quantity estimation. Each question scores as 0 (unhealthy) or 1 (healthy), and the final score undergoes a classification procedure. Through the framework of a nutritional study, 152 employees of the municipal water company in Guadalajara, Mexico (April–August 2016), were invited to participate. The survey was administered in two rounds (test and retest) with a 15-day interval between them. We calculated the Spearman correlation coefficient, the intra-class correlation coefficient (ICC), and weighted kappa for score classification agreement (SPSS versus 14 *p* < 0.05 was considered statistically significant). The survey obtained a “good” reproducibility (ρ = 0.713, *p* < 0.001), and an excellent concordance (ICC = 0.841 Confidence Interval 95% 0.779, 0.885). It can thus be said that the Mini-ECCA displayed acceptable reproducibility and is suitable for the purpose of dietary assessment and guidance.

## 1. Introduction

The concept of dietary quality includes categories such as healthy diet, balanced diet, nutritious foods, functional foods, and optimal nutrition [1]. It also encompasses sociocultural, organoleptic, and food safety aspects. To evaluate dietary quality, specific tools, such as surveys or indices, are available for different populations [2]. Such tools compare foods and nutrients actually consumed with those that have been recommended, and make it possible to categorize individual subjects in accordance with their dietary patterns [2,3]. These indices are based on the analysis of survey-type tools, such as 24-h recalls, food records, and food frequency questionnaires. Some of the most popular of these tools are the Healthy Eating Index (HEI) [4], the Diet Quality Index (DQI) [5], and the Healthy Diet Index (HDI) [6], in addition to other indices that have been adapted from these [2]. To the best of our knowledge, only one validated and reproducible tool for the analysis of dietary quality (ICDMx) currently exists in Mexico [3]. The ICDMx is based on energy and nutrient intake results from food frequency questionnaires and dietary records. It is comprised of five categories, which assess whether the diet is sufficient, balanced, complete, varied, and innocuous. Another tool had previously been developed, which consisted of three dietary quality indices: (1) a cardioprotective index, which assessed adherence to seven World Health Organization (WHO) recommendations related to the intake of protein, fat, different types of fatty acids, etc.; (2) a micronutrient adequacy index for calcium, iron, zinc, folate, vitamin A, and vitamin C, based on the estimated average requirements from the U.S. Institute of Medicine; and (3) a dietary diversity index, based on the consumption of 30 food groups, such as tortilla, legumes, whole cereal products, etc. However, these indices were not validated [7].

One of the main challenges associated with diet quality indices is that they are time-consuming (in terms of administration, data analysis, and interpretation) and require well-trained interviewers. In this regard, other tools to assess food intake that are more focused on evaluating the adherence to specific intake recommendations are available—in particular, tools associated with the Mediterranean diet [8,9,10,11,12,13,14,15]. These instruments have the advantage of short administration times, as well as highly accurate and faster interpretations of the intake of certain foods or food groups. However, the reproducibility and validity of data are generally not available for these instruments, with the exception of reproducibility data in the case of the Mediterranean Diet Quality Index (KIDMED) [15].

Because food is a determinant factor in the development of non-communicable chronic diseases [16,17,18,19,20], knowledge of food intake quality and adherence to food intake recommendations is important in the field of public health, since it enables the development of various food aid programs, the enactment of fortification and labeling policies, and the preparation of nutritional recommendations [19]. Nonetheless, in Mexico there are no similar tools for the assessment of adherence to food recommendations that have been scientifically screened, are easy and quick to administer, and offer the possibility of giving feedback easily and accurately to the population. For this reason, this study aims to describe the design and assess the reproducibility (reliability) of a mini-survey to evaluate the quality of food intake in a Mexican population (called Mini-ECCA because of its initials in Spanish: Mini Encuesta de Calidad de Consumo Alimentario), using the test–retest method.

This study is part of the “Comprehensive Health Care Program: APAIS-SIAPA” project led by Barbara Vizmanos. The project was developed in response to a special request by the Guadalajara (Mexico) municipal water company Sistema Intermunicipal de Servicios de Agua Potable y Alcantarillado (SIAPA). The person co-responsible for the University of Guadalajara’s nutrition program is Fabiola Márquez Sandoval. Within this program, the sub-project “The creation, validation and administration of a mini survey to assess the quality of food intake (Mini-ECCA) in a Mexican population” was implemented under the leadership of María Fernanda Bernal Orozco, Gabriela Macedo Ojeda, and Barbara Vizmanos. This paper presents reproducibility data for the Mini-ECCA.

## 2. Materials and Methods

### 2.1. Population and Study Design

A reproducibility study was conducted from April to August 2016, on a convenience sample of male and female volunteers aged 18 years or older who worked for the municipal water company SIAPA in the Guadalajara Metropolitan Area (Jalisco, Mexico), and who were participants in the above-mentioned project. Subjects included administrative staff, service staff, operational staff, engineers, managers, and other workers. All subjects participated on a voluntary basis, signed an informed consent form, and were able to read and write. Pregnant or lactating women were not included, owing to the particular nature of the food habits and requirements associated with this life stage. Also excluded were individuals who did not take the Mini-ECCA a second time, and those who declined to participate in spite of having signed the informed consent.

In accordance with criteria found in scientific literature, studies of reproducibility and validity should include approximately 10 subjects for each item in the evaluated survey [21]. Hence, the minimum sample in this study would necessarily have 120 subjects.

With respect to ethical considerations, the Declaration of Helsinki guidelines for human research were followed in order to ensure respect for the dignity, rights, and well-being of participants, in accordance with ethical and scientific principles. Furthermore, data confidentiality was guaranteed [22] and the study’s protocol was approved by the university’s Ethics, Research and Biosafety Committees (registry number: 02216).

### 2.2. Design of the Mini-ECCA Questionnaire

The Mini-ECCA is comprised of 12 items, developed based on Mexican and international recommendations (Table 1) and a review of other relevant publications. In particular, the Mexican Diet Quality Index (ICDMx) served as the basis for the development of this survey [3]. In addition, a validation of these recommendations was carried out by a group of experts (six health professionals with both clinical and research experience).

For each question in the survey, visual aids in the form of photographs are used to estimate food quantities (see Presentation S1: Mini-ECCA’s visual aid for food quantity estimation). Focused on food and non-alcoholic beverages, the survey is quick to administer (5 min). For each item, one point is given when the intake recommendations of a certain food group or subgroup are met. When recommendations are not met, 0 points are given. The maximum possible score is 12 points. Based on the final score, food intake quality can be classified as follows: very good (10–12), good (7–9), low (4–6), and very low (1–3) (see Table S1: Mini-ECCA survey).

### 2.3. Data Collection Procedures and Strategies

Following coordination with SIAPA’s management and medical staff, a series of visits by researchers to the company’s offices was planned, during which employees would be evaluated as subjects using the Mini-ECCA.

A total of two visits were made for each employee. Two weeks prior to the first visit to the SIAPA offices, invitation posters were put up in key areas frequented by the majority of employees. These posters, as well as cards that were also distributed, contained basic information about the nutrition project and stated the date and time of the first visit. The activities of each visit are outlined below:

Visit 1: Individuals interested in participating were called to a session at which one of the researchers (Montserrat González Gómez) explained the project’s objective, the importance of their participation, what would be expected of them, and the benefits and significance of their participation. The volunteer subjects signed an informed consent form and filled out a personal data sheet, which included a subjective evaluation of their physical activity level. They filled out these forms under the personalized guidance of previously trained evaluators. An anthropometric assessment was then performed by an anthropometrist specially trained for this evaluation (a total of two anthropometrists were trained). Measurements were taken and recorded by a scorer. The measurements were for height (using a SECA^®^ stadiometer, SECA GMBH & Co., Hamburg, Germany; model 213, accurate to 0.1 cm, measuring range up to 230 cm), body weight, body fat percentage, total body water, visceral fat, muscle mass, bone mass (using a TANITA^®^ scale, Tanita Corporation, Tokyo, Japan; model BC 558, 150 kg capacity, accurate to 0.1 kg), and waist and hip circumference (using a Lufkin Rosscraft^®^ metal tape measure, Apex Tool Group, Sparks, NV, USA; model W606, range of 0 to 200 cm, accurate to 0.1 cm). Weight, height, and circumferences were measured according to ISAK (International Society for the Advancement of Kinanthropometry) standards [34]. Body mass index (BMI) was also calculated and classified, based on criteria proposed by the World Health Organization [35]. The Mini-ECCA was then administered to each participant by another trained evaluator. Lastly, a final evaluator scheduled each participant for his or her next appointment. A total of five evaluators performed each of the above-described functions in rotation, except in the case of the two anthropometrists.

Visit 2: On this visit, 15 days after the first visit (enough time had passed for the subjects not to remember what their previous answers had been exactly; however, not enough time had passed for them to have significantly changed their diet) [36,37], the Mini-ECCA was administered for the second time, by the same evaluator who had administered the survey on the previous visit. It should be noted that there were no important celebrations or holidays during the interval between administrations that could have given rise to changes in the participants’ habitual food intake. As a form of compensation for participating in this study, each participant was given feedback on their survey results. In addition, they were given suggestions on the habits they should improve on and strategies to achieve these improvements. They were also offered the opportunity to work on a specific nutritional goal (chosen by the worker) for the next 30 days, after which their efforts would be evaluated.

### 2.4. Statistical Analysis

Qualitative variables are presented as frequency (percentage), and quantitative variables as mean ± standard deviation. The average and standard deviation of the total score of each Mini-ECCA were calculated. In addition, the number and proportion of participants who obtained 1 or 0 points on each of the Mini-ECCA’s items were determined.

The reproducibility of the Mini-ECCA’s total score was determined using a linear Spearman correlation between both Mini-ECCAs. In addition, agreement between test and retest scores was evaluated using the intra-class correlation coefficient (%, confidence interval). Significant changes in the test and retest scores were also analyzed using the McNemar test (lack of significance means that no changes were observed between test and retest). In addition, the capacity to classify individuals was explored, using the agreement between the answers of each question in the Mini-ECCA, as well as with the categories of the total score with Cohen’s kappa and weighted kappa—which ranges from −1 (perfect disagreement) to 1 (perfect agreement)—and categorized according to Landis and Koch criteria [38].

For the statistical analyses, SPSS (version 14, Inc. SPSS, Chicago, IL, USA, 2006) and Microsoft Office Excel 2010 for Windows 8.1 (version 14, Microsoft, Redmond, WA, USA, 2010) were used.

## 3. Results

### 3.1. Description of the Population

When the study began, 304 subjects agreed to participate. Of these, 98 did not attend the second visit (the Mini-ECCA was administered only once). A total of 206 subjects completed the study (98 men and 108 women).

The average age was 40.5 ± 8.1 for men and 41.3 ± 7.1 for women. Most subjects were sedentary, had a bachelor’s degree, and worked as administrative staff (Table 2). In addition, four (1.9%) were diabetic, 28 (13.6%) had high blood pressure, four (1.9%) reported having another type of cardiovascular disease, four (1.9%) reported having cancer, and 69 (33.5%) reported having other disorders, mainly gastritis (12.6%) and colitis (8.7%) (data not shown). When comparing general and anthropometric characteristics between the included subjects and those withdrawn from the study, excluded subjects were mostly men (65.3% versus 47.6% in included subjects; data not shown).

With respect to the population’s anthropometric characteristics, men were found to have significantly higher values than women for height, body weight, BMI, body water percentage, visceral fat, bone mass, muscle mass, and waist circumference. However, the percentage of body fat was higher in women. In addition to the above, the majority of participants (77.2%) were overweight according to the WHO’s [35] classification (Table 3).

### 3.2. Reproducibility and Concordance of the Mini-ECCA

**Figure 1:**
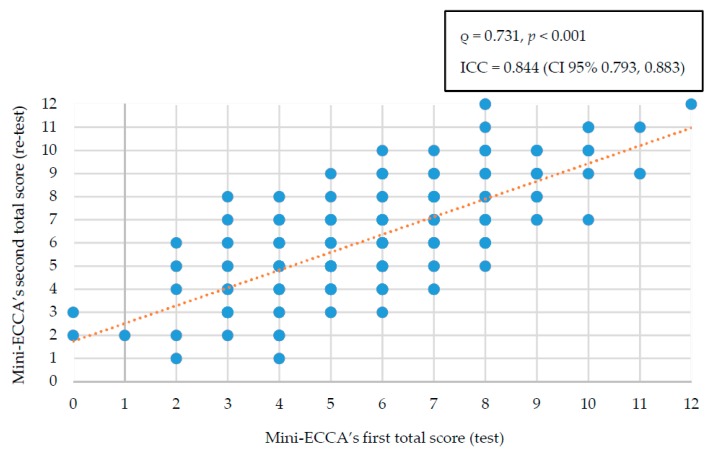
Reproducibility of the total scores obtained in the test–retest. ICC: intra-class correlation coefficient. CI: confidence interval.

The average total score from the Mini-ECCA’s first administration was 6.1 ± 2.2 out of 12 possible points, while on the second administration it was 6.4 ± 2.3. The correlation between both scores was ρ = 0.731, *p* < 0.001. Likewise, when the concordance of the total score between both administrations was analyzed, an intra-class correlation coefficient of 0.844 (95% CI 0.793, 0.883) was obtained (Figure 1).

Following the first administration of the Mini-ECCA, 3.9% of the sample was within the 10–12 point range, and hence classified as having a high level of food intake quality. However, after the second administration, this value increased to 7.2%. It was also observed that, irrespective of initial classification, scores worsened for 13.6% of the population in the retest and improved for 24.3% (data not shown). A moderate level of agreement in the food intake quality classification was thus observed between both administrations of the Mini-ECCA (weighted *κ* = 0.545, 95% CI 0.484, 0.606, *p* < 0.001) (Table 4).

When evaluating concordance of the answers for each question between both survey administrations, agreement between the majority of the variables (9 of 12) was moderate (0.40–0.59), and in the case of cereals it was very low (<0.40). However, some questions showed significant changes in the retest answers compared to the first test. These included those that mentioned fish, sweetened beverages, foods not prepared at home, legumes, and the most frequently consumed meats. The results for each question are shown in Table 5 for dichotomous responses, Table 6 for cereals, and Table 7 for fats and meats.

## 4. Discussion

The Mini-ECCA is the first short screening tool in Mexico that evaluates food intake quality with a focus on food and non-alcoholic beverages, and which also enables a classification of this characteristic to be performed. In this study, reproducibility was evaluated using the test–retest method, with a 15-day interval between administrations. The tool provided a good level of reproducibility, excellent concordance in the final score, and moderate concordance in the classification of food intake quality.

The scientific literature mentions tools that are very similar in content to the Mini-ECCA, but which do not evaluate the concept of food intake quality. Instead, they assess adherence to intake recommendations, and in particular adherence to the Mediterranean diet. Among them are the MEDI-LITE Score [14], the Chilean Index of the Mediterranean Diet (Índice Chileno de la Dieta Mediterránea, IDM-Chile) [39], the Mediterranean Dietary Serving Score (MDSS) [13], the Mediterranean Diet Assessment Tool [12], the Mediterranean Diet Adherence Screener (MIDAS) [11], the Mediterranean Dietetic Quality Index (Med-DQI) [8], the Mediterranean Style Dietary Pattern Score (MSDPS) [9], and the Med Diet Score (MDS) [10]. Other tools evaluate the Mediterranean diet’s association with diseases. They include EVIDENT [40], ITALIAN-MED [41], the Mediterranean Relative Diet (rMED) [42], the Cardioprotective Mediterranean Diet Index (CARDIO) [43], and the Japanese-adapted MD score (jMD score) [44].

Of these, the only tool for which reproducibility data were found is the Mediterranean Diet Quality Index (KIDMED). Štefan et al. evaluated this tool’s reproducibility in a university student population (mean age 19.7 ± 1.3 years), using the test-retest method, with intervals of 15 days between administrations. Reproducibility was determined using a concordance analysis for each question and from the classification of adherence to the Mediterranean diet (poor, average, or good) that was obtained based on the total score. In this regard, the authors obtained a good level of agreement for the adherence classification (*κ* = 0.597, *p* < 0.001) [15], and these results were similar to those obtained using the Mini-ECCA (weighted *κ* = 0.545, 95% CI, 0.484 to 0.606).

Regarding the agreement between the answers for each question across both administrations of the Mini-ECCA, agreement for the majority of the questions (9 of 12) was moderate (0.41–0.60), while that for questions referring to the intake of natural water and sweetened beverages was good (0.61–0.80). Only the question covering intake preferences for the different types of cereals showed a weak concordance (<0.40), with the observation that, on the second administration, a greater number of the subjects (6 of 8) who answered “Do not know” the first time answered that they consumed “whole grain” cereals on the second administration. It is possible that the use of visual aids on the first administration of the Mini-ECCA sensitized participants, and that following the first administration the participants who did not know what type of cereal they were consuming would pay more attention and respond in a more complete way on the second. Another possibility is that those who realized that they were consuming “refined” instead of “whole grain” cereals modified their intake, or chose to give “whole grain” as an answer for reasons of social prestige. There is also a possibility that the inclusion of the “Do not know” response option created confusion that affected the ability of subjects to distinguish between different types of cereals, and thus favored a change in the responses given between the first and second administrations of the Mini-ECCA.

Likewise, although agreement was moderate for the question about fish, it was the question for which the largest proportion of subjects gave a different response in the retest. A total of 16% of the participants contributed to an increase in the score of this response in the second administration. In a way similar to question about cereals, it is possible that participants became sensitized, and thus increased their intake in the weeks following the first administration. No feedback was provided to participants regarding their responses on the first administration of the Mini-ECCA, as they were told that this would take place during the second administration.

In the KIDMED reproducibility study, all questions showed moderate to excellent agreement in their answers (*κ* between 0.504 and 0.849). However, the proportion of subjects who responded affirmatively to the question about consuming pasta or rice at least five times a week increased significantly in the retest (McNemar test, *p* = 0.016). In this study, responses to the question about the intake of vegetables more than once per day showed the lowest concordance (*κ* = 0.505) [15].

It should also be noted that the Mini-ECCA makes it possible to both determine a total score for food intake and to make a classification. In both administrations, the largest proportion of the study population (30.3% in the first administration and 27.6% in the second) obtained 4–6 points, and was classified in the “low” food intake quality category. This result is similar to that reported in the KIDMED reproducibility study, where in both administrations the highest proportion of the population (approximately 40%) was classified in the “average” category (4–7 points) [15]. It should be noted that, although there were no significant changes in the responses given in the retest compared to the initial test, 24 of the 92 subjects initially classified in the “low” food quality category (26.1%) improved the quality of their food intake in the retest, positioning themselves in the “good” (7–9 points, *n* = 23) or “very good” food intake quality (10–12 points, *n* = 1) classifications. Conversely, 15 of the 77 subjects who initially scored in the “good” intake quality category (19.5%) worsened their quality in the retest, to the point of falling into the “low” intake quality category. It is difficult to determine the specific changes in habits that could have resulted in this food intake shift. In the case of subjects whose food intake quality improved, the questions that showed the largest score increases were those about the intake of meat (8 of 77, 10%), vegetables, sweets, and cereals (6 of 77 or 8% in all three cases). In contrast, in cases where food intake quality worsened, the questions showing the greatest test versus retest score decreases were those about the intake of sweets (6 of 15, 40%), processed foods and cereals (5 of 15 or 33% in both cases) (data not shown).

In regards to the intake habits detected by Mini-ECCA, approximately 40% of subjects did not consume 1.5 L of water or a minimum of 200 g of fruit per day; 40–50% did not consume a minimum of 100 g of fish per week, consuming mainly beef instead; more than 50% did not consume a minimum of 200 g of vegetables per day. These same subjects frequently consume foods prepared outside the home and processed foods, and they do not consume monounsaturated fats as their main source of fat. In addition, more than 60% consume sweets or desserts two or more days per week, and more than 70% report eating legumes on a daily basis, consuming mainly whole grains and drinking more than four sweetened beverage drinks per week. In short surveys that evaluate dietary quality and adherence to the Mediterranean diet in healthy adults from different countries, habits similar to those reported in the Mini-ECCA were observed [15,45]. These habits included a low intake of vegetables, grains, meat, or protein alternatives like legumes [46]. These data should be a cause for concern for the Mexican population, given that—as we have previously stated—food is a key factor in the development of a range of chronic non-communicable diseases.

In addition, some of these data are consistent with the findings of the 2016 National Health and Nutrition Survey of Medio Camino (ENSANUT MC), a nationally representative study of the health and nutritional status of the Mexican population. The sex distribution reported in this study (women = 51.1%) was similar to that of ours, while educational levels (elementary school = 32.6%; professional studies = 9.3%; postgraduate < 1%) were lower. In that study, the high prevalence of overweight and obese subjects (77.2%) was similar to that reported in our survey (72.5%), although in our case this prevalence was higher among men (88.7% versus 69.5%) and lower among women (67.3% versus 75.6%). In addition, the previous survey found that, according to a food frequency questionnaire, 47% of the population consumed fruits and vegetables on a daily basis (less than our study) [47], while other studies have reported that less than 30% of the Mexican population complies with the recommended intake of 400 g per day, consuming instead an average daily amount of 122.6 g [48]. Furthermore, 70% consume legumes three or more times per week (as in our study), and a high proportion of subjects frequently consume unhealthy foods (as in our sample) such as non-dairy sweetened beverages (85.3%); sweetened milk drinks (24.1%); fast food and Mexican snack foods (18.3%); snacks, sweets, and desserts (38%); processed meats (19.8%); and sweet cereals (45.6%) [47]. A high intake of sweetened beverages coupled with a low intake of water has already been found in other studies [49,50,51], as have low levels of fish intake [52], a high intake of processed foods [53], and a high frequency of eating outside the home [54]. With these data, it can be observed that our results are consistent with those described in other studies, and could be applicable in the general population, in spite of differences in educational level, nutritional status, and methods for assessing food intake.

One limitation of this study is that, due to the low number of items, a one-point variation between test and retest scores will have an impact on the food intake quality classification, which could cause an average value to become a high value, or a medium value to change to a low one. It may therefore be worthwhile to consider changing the Mini-ECCA’s response option format from a dichotomous one to the type found on a Likert scale. Another limitation is that reproducibility was evaluated after an interval of 15 days. Although this same period between test and retest has been reported in other studies [15], it would be interesting to analyze reproducibility after a longer interval (30 days), and evaluate the performance of the questions that show a lower level of agreement between administrations. Furthermore, a number of tools that evaluate dietary quality and adherence to a diet include alcohol intake [3,10,12,13,55,56,57,58]. The initial focus of the Mini-ECCA was on food and non-alcoholic beverages. Nonetheless, given that alcohol intake in Mexican adults has increased by 14.2% over the last 12 years—a situation that resulted in 69.8 deaths per 100,000 inhabitants in 2012 in Mexico [59]—alcohol intake should be considered as a factor to be evaluated in future versions of the Mini-ECCA. Another possible limitation of our survey is that some questions may not be easily understood by people unfamiliar with nutritional terminology. In such cases, providing verbal explanations of questions may improve response accuracy. In addition, since children and young adults were not among this study’s participants, the use of this instrument on such populations would require prior assessment. Another potential issue, which may appear in any type of food record, is that some respondents may state that they are healthier than they actually are, particularly in the retest phase.

One of our survey’s strengths is its use of visual support (photographs), a factor which decreases the risk of overestimating or underestimating food intake amounts. Another strength is its short length (12 items), which makes short administration times possible (approximately five minutes). It is also a tool that uses suggested minimum intake quantities (fruits, vegetables, water, legumes, and fish) or maximum quantities (sweetened beverages, desserts, processed foods, and foods prepared outside the home) in each of its questions, with the aim of obtaining data of a specific nature. In addition, the visual aids we used make it possible to obtain estimates that reflect the quantities stipulated in the tool more accurately than might otherwise be the case, since the images show the actual size of the portions in question, and help to differentiate the foods that make up each group. In addition, the Mini-ECCA could be used by health professionals who need a quick way to assess dietary habits, or foster improvements in such habits within a particular population. Finally, the survey could be used to help set goals and monitor progress towards achievement when providing nutritional care to a range of patients.

With respect to future use of this survey, it will be important to assess its performance with other populations, such as children, adolescents, and older adults. It may also be worthwhile to analyze its usefulness in a clinical context, such as that of providing nutritional guidance, as questions from the Mini-ECCA could be used to establish nutritional goals.

## 5. Conclusions

The Mini-ECCA is a short screening tool for the evaluation of food intake quality, based on national and international recommendations. It is also focused on food and non-alcoholic beverages, and requires visual aids during administration. In a population of working adults, this tool showed good reproducibility and excellent concordance in the final score, while concordance in the classification of food intake quality was moderate. As a result, its administration to adult Mexican populations is feasible, since no other tools of a similar nature are currently available.

## Figures and Tables

**Table 1:** Recommendations used to prepare the Mini-ECCA and questions, which were developed based recommendations (images and options are shown simultaneously in a picture when asking the Mini-ECCA questions).

Food Group/Food	Evidence	Mini-ECCA Question	Reference
Water	An intake of 1.5 L of water per day is recommended, as it facilitates the proper functioning of various organic processes, such as temperature regulation and the elimination of metabolites. Water also plays a role in the processes of digestion, absorption, and excretion, and it serves as a means of distributing nutrients.	Do you drink at least 1.5 L of water per day?	[3,23]
Vegetables	At least 200 g of vegetables per day are recommended. They are a good source of vitamins, minerals, and fiber, and also have low energy density. As the WHO ^1^ recommends a 400 g combined intake of vegetables and fruits, fruits as a single group were assigned a minimum recommendation of half that amount (200 g).	Do you consume at least 200 g of cooked or raw vegetables per day?	[24]
Fish	In general, fish is lower in saturated fat and higher in polyunsaturated fats (Omega-3) than other sources of animal protein. The American Heart Association recommends two servings of fish per week, and that each serving should be 3.5 ounces (100 g). However, because fish intake levels in Mexico are low (6.7% of all proteins consumed) [25], it was decided that this food type should be consumed at least once per week (100 g) as a minimum goal.	Do you eat fresh or frozen fish (100 g) at least one day per week?	[26]
Types of meat	A high intake of red meat has been linked to the development of colorectal cancer. In addition, it contains higher levels of saturated fat than other meats. For this reason, the intake of red meat should be minimized in favor of other animal products, such as chicken and fish.	What type of meat do you consume most often?	[27,28]
Types of fat	The recommended intake of fatty acids is less than 7% of the total energy expenditure for saturated fat, 6–10% in the case of polyunsaturated fat, and the difference between total fat minus the latter two recommendations, in the case of monounsaturated fat. Adherence to these percentages is considered crucial for the prevention of cardiovascular diseases and their risk factors.	What type of fat do you most frequently consume during the week?	[3,29,30]
Sweetened beverages	Evidence exists to establish an association between the increase in the prevalence of overweightness, obesity, and diabetes in Mexico in the last 20 years, and the increase in the intake of kilocalories from sweetened beverages in the same period. Based on this evidence and current WHO recommendations regarding the intake of sugars (a maximum of 10% and preferably less than 5% of total energy intake), it is recommended that sweetened beverage intake should not exceed one drink (250 mL) per day. However, due to the high intake of sugar-sweetened beverages in the population, we agreed on an intermediate limit (four sugar-sweetened beverages per week).	Do you consume four or more sweetened beverages per week?	[31,32]
Fruits	A minimum intake of 200 g of fruit per day is recommended. Fruits are a good source of vitamins, minerals, antioxidants, and fiber. As the WHO recommends 400 g of vegetables and fruits combined, fruits as a single group were assigned a minimum recommendation of half that amount (200 g).	Do you consume at least 200 g of fruit per day?	[33]
Foods consumed outside the home	People who consume food outside the home have a higher risk of being overweight or obese, due to the amount of energy contained in such foods, and because the portions consumed outside the home tend to be larger than those consumed at home.	Do you eat foods not prepared at home three or more days per week?	[28,31,33]
Processed foods	Many processed foods contain high amounts of sodium. In this regard, the WHO recommends reducing sodium intake in adults to less than 2 g/day (5 g/day of salt). The WHO has also said that the intake of processed meat should be limited, because it has been linked to cancer.	Do you eat processed foods two or more days per week?	[27,31,32,33]
Desserts	Desserts contain high amounts of fats and sugars. In this regard, the WHO recommends reducing the intake of free sugars to less than 5% of the total daily calories, based on evidence for the development of various illnesses, including dental caries. The consensus of experts has determined that these foods should not be completely eliminated. Instead, healthy habits should be encouraged and the recommended intake frequency from this category should be no more than three days per week.	Do you consume sweets or commercial desserts two or more days per week?	[28,31,33]
Legumes	Intake from this food group is encouraged, owing to its fiber and protein content. Recommendations call for a minimum intake of three times per week. Due to the variability of the quantities cited in the various information sources (225–450 g), experts recommend a minimum quantity of 300 g per week.	Do you eat legumes at least three days per week (300 g per week)?	[3,11,12,28]
Cereals	It is recommended that 50% of cereals consumed be whole grain, due to their higher fiber content. Fiber is known to improve digestion, and to decrease blood glucose and cholesterol levels.	What cereals do you consume most often?	[28]

^1^ WHO (World Health Organization). Mini-ECCA: Mini-Survey to Evaluate the Quality of Food Intake.

**Table 2:** General characteristics of the population (*n* = 206).

Variable	Frequency	Percentage
Sex		
Male	98	47.6
Female	108	52.4
Physical activity		
Sedentary lifestyle	76	36.9
Light	62	30.1
Moderate	68	33.0
Education		
Postgraduate	13	6.3
Bachelor’s degree	78	37.9
High school	69	33.5
Middle school	40	19.4
Elementary school	6	2.9
Work area		
Administrative	118	57.3
Operations	88	42.7

**Table 3:** Anthropometric characteristics of the population.

Variable	Men (*n* = 98)	Women (*n* = 108)	Total (*n* = 206)
Age	40.5 ± 8.1	41.3 ± 7.1	40.9 ± 7.6
Height (cm)	171.8 ± 6.1 ***	160.7 ± 5.9	166.0 ± 8.2
Weight (kg)	86.8 ± 14.7 ***	71.3 ± 13.2	78.7 ± 15.9
Body mass index (kg/cm^2^)	29.4 ± 4.5 **	27.6 ± 5.0	28.4 ± 4.8
BMI classification (WHO)			
Normal	11 (11.2)	36 (33.3)	47 (22.8)
Overweight	50 (51.0) **	41 (38.0)	91 (44.2)
Class I Obesity	28 (28.6)	22 (20.4)	50 (24.3)
Class II Obesity	7 (7.1)	7 (6.5)	14 (6.8)
Class III Obesity	2 (2.0)	2 (1.9)	4 (1.9)
Body fat percentage (%)	26.5 ± 5.6	37.0 ± 6.2 ***	31.9 ± 7.9
Body water percentage (%)	52.5 ± 4.1 *** ^1^	46.5 ± 4.2	49.3 ± 5.1 ^2^
Percentage of visceral fat (%)	11.1 ± 4.2 ***	6.9 ± 2.9	8.9 ± 4.2
Bone mass (kg)	3.2 ± 0.4 ***	2.3 ± 0.3 ^3^	2.7 ± 0.5 ^4^
Muscle mass (kg)	60.1 ± 7.4 *** ^5^	42.1 ± 5.1 ^3^	50.7 ± 11.0 ^6^
Waist circumference (cm)	97.3 ± 11.6 ***	84.8 ± 11.2	90.7 ± 13.0
Hip circumference (cm)	102.6 ± 7.4 ^5^	104.9 ± 9.9	103.8 ± 8.9 ^4^

Values are presented as an average ± standard deviation or frequency (percentage). ** Unpaired *t*-test for quantitative variables and chi-squared test for qualitative variables, *p* < 0.01; *** *p* < 0.001. ^1^ *n* = 96; ^2^ *n* = 204; ^3^ *n* = 107; ^4^ *n* = 205; ^5^ *n* = 97; ^6^ *n* = 204.

**Table 4:** Concordance of the food intake quality classification, according to the total Mini-ECCA score (*n* = 206).

Classification According to the Scores Obtained		Test					McNemar Test (*p*-Value)	Weighted *** (95% CI)
		Very Low (1–3 points)	Low (4–6 points)	Good (7–9 points)	Very Good (10–12 points)	Total ***		
**Retest**	Very low	**13 (52.0)**	9 (9.8)	0 (0.0)	0 (0.0)	22 (7.2)	0.091	0.545 (0.484, 0.606)
	Low	10 (40.0)	**59 (64.1)**	15 (19.5)	0 (0.0)	84 (27.6)		
	Good	2 (8.0)	23 (25.0)	**49 (63.6)**	4 (33.3)	78 (25.7)		
	Very good	0 (0.0)	1 (1.1)	13 (16.9)	**8 (66.7)**	22 (7.2)		
	Total	25 (8.2)	92 (30.3)	77 (25.3)	12 (3.9)	206 (100.0)		

Data are presented as frequency (percentage of test subjects who showed agreement on the retest; total agreement is shown in boldface). *** *p* < 0.001. CI: confidence interval.

**Table 5:** Agreement between Mini-ECCA questions with dichotomous responses (*n* = 206).

Variable	Retest	Test			McNemar Test (*p*-Value) ^1^	Kappa *** (95% CI)	Test vs. Retest Score Differences		
		Yes	No	Total			Decreased	Remained Unchanged	Increased
1. Do you drink at least 1.5 L of water per day?	Yes	**104 (89.7)**	24 (26.7)	128 (62.1)	0.065	0.639 (0.533, 0.744)	12 (5.8)	170 (82.5)	24 (11.7)
	No	12 (10.3)	**66 (73.3)**	78 (37.9)					
	Total	116 (53.3)	90 (43.6)	206 (100)					
2. Do you consume at least 200 g of cooked or raw vegetables per day?	Yes	**69 (78.4)**	28 (23.7)	97 (47.1)	0.243	0.540 (0.424, 0.655)	19 (9.2)	159 (77.2)	28 (13.6)
	No	19 (21.6)	**90 (76.3)**	109 (52.9)					
	Total	88 (42.7)	118 (57.3)	206 (100)					
3. Do you eat fresh or frozen fish (100 g) at least one day per week?	Yes	**87 (88.8)**	33 (30.6)	120 (58.3)	**<0.001**	0.576 (0.468, 0.683)	11 (5.3)	162 (78.6)	33 (16.0)
	No	11 (11.2)	**75 (69.4)**	86 (41.7)					
	Total	98 (47.6)	108 (52.4)	206 (100)					
4. Do you consume four or more sweetened beverages per week?	Yes	**143 (87.7)**	7 (16.3)	150 (72.8)	**0.019**	0.643 (0.521, 0.764)	7 (3.4)	179 (86.9)	20 (9.7)
	No	20 (12.3)	**36 (83.7)**	56 (27.2)					
	Total	163 (79.1)	43 (20.9)	206 (100)					
5. Do you consume at least 200 g of fruit per day?	Yes	**107 (79.3)**	19 (26.8)	126 (61.2)	0.243	0.510 (0.388, 0.631)	28 (13.6)	159 (77.2)	19 (9.2)
	No	28 (20.7)	**52 (73.2)**	80 (38.8)					
	Total	135 (65.5)	71 (34.5)	206 (100)					
7. Do you eat foods not prepared at home three or more days per week?	Yes	**100 (87.0)**	29 (31.9)	129 (62.6)	**0.049**	0.560 (0.446, 0.674)	29 (14.1)	162 (78.6)	15 (7.3)
	No	15 (13.0)	**62 (68.1)**	77 (37.4)					
	Total	115 (55.8)	91 (44.2)	206 (100)					
9. Do you eat processed foods two or more days per week?	Yes	**84 (81.6)**	31 (30.1)	115 (55.8)	0.119	0.515 (0.399, 0.631)	31 (15.0)	156 (75.7)	19 (9.2)
	No	19 (18.4)	**72 (69.9)**	91 (44.2)					
	Total	103 (50.0)	103 (50.0)	206 (100)					
10. Do you consume sweets or commercial desserts two or more days per week?	Yes	**107 (79.3)**	23 (32.4)	130 (63.1)	0.576	0.461 (0.335, 0.586)	23 (11.2)	155 (75.2)	28 (13.6)
	No	28 (20.7)	**48 (67.6)**	76 (36.9)					
	Total	135 (65.5)	71 (34.5)	206 (100)					
11. Do you eat legumes at least three days per week (300 g per week)?	Yes	**144 (93.5)**	24 (46.2)	168 (81.6)	**0.024**	0.520 (0.381, 0.659)	10 (4.9)	172 (83.5)	24 (1.7)
	No	10 (6.5)	**28 (53.8)**	38 (18.4)					
	Total	154 (74.8)	52 (25.2)	206 (100.0)					

Data are presented as frequency (percentage of test subjects who showed agreement on the retest; total agreement is shown in boldface). ^1^ McNemar test *p*-values in boldface are significant. *** *p* < 0.001.

**Table 6:** Concordance for the question on cereals with three response options from the Mini-ECCA (*n* = 206).

Variable	Retest	Test				McNemar Test (*p*-Value)	Weighted Kappa *** (95% CI)	Test vs. Retest Score Differences		
		Whole Grain	Refined	Do Not Know	Total			Decreased	Remained Unchanged	Increased
12. What cereals do you consume most often?	Whole grain	**148 (90.2)**	20 (58.8)	6 (75.0)	174 (84.5)	0.435	0.271 (0.218, 0.324)	16 (7.8)	164 (789.6)	26 (12.6)
	Refined	12 (7.3)	**12 (35.3)**	1 (12.5)	25 (12.1)					
	Do not know	4 (2.4)	2 (5.9)	**1 (12.5)**	7 (3.4)					
	Total	164 (79.6)	34 (16.5)	8 (3.8)	206 (100.0)					

Data are presented as frequency (percentage of test subjects who showed agreement on the retest; total agreement is shown in boldface). *** *p* < 0.001.

**Table 7:** Agreement between both Mini-ECCA questions (fats and meats) with four response options (*n* = 206).

Variable	Retest	Test					McNemar Test (*p*-Value) ^1^	Weighted Kappa *** (95% CI)	Test vs. Retest Score Differences		
		A	B	C	D	Total			Decreased	Remained Unchanged	Increased
6. What type of fat do you most frequently consume during the week?	A (Polyunsaturated)	**69 (76.7)**	14 (14.9)	6 (42.9)	3 (37.5)	92 (44.7)	0.204	0.456 (0.397, 0.515)	15 (7.3)	171 (83.0)	20 (9.7)
	B (Monounsaturated)	14 (15.6)	**79 (84.0)**	1 (7.1)	5 (62.5)	99 (48.1)					
	C (Saturated)	2 (2.2)	1 (1.1)	**6 (42.9)**	0 (0.0)	9 (4.4)					
	D (Do not know)	5 (5.6)	0 (0.0)	1 (7.1)	**0 (0.0)**	6 (2.9)					
	Total	90 (43.7)	94 (45.6)	14 (6.8)	8 (3.9)	206 (100.0)					
8. What type of meat do you consume most often?	A (Chicken)	**75 (81.5)**	22 (24.2)	2 (25.0)	6 (40.0)	105 (51.0)	**0.013**	0.460 (0.399, 0.521)	14 (6.8)	164 (79.6)	28 (13.6)
	B (Beef)	8 (8.7)	**69 (75.8)**	0 (0.0)	6 (40.0)	83 (40.3)					
	C (Fish)	3 (3.3)	0 (0.0)	**6 (75.0)**	0 (0.0)	9 (4.4)					
	D (Do not know)	6 (6.5)	0 (0.0)	0 (0.0)	**3 (20.0)**	9 (4.4)					
	Total	92 (44.7)	91 (44.2)	8 (3.9)	15 (7.2)	206 (100)					

Data are presented as frequency (percentage of test subjects who showed agreement on the retest; total agreement is shown in boldface). ^1^ McNemar test *p*-values in boldface are significant. *** *p* < 0.001.

## Supplementary Materials

The following are available online at http://www.mdpi.com/2072-6643/10/4/524/s1, Presentation S1: Mini-ECCA’s visual aid for food quantity estimation, Table S1: Mini-ECCA survey.
