# Supplementary material for: Design and Reproducibility of a Mini-Survey to Evaluate the Quality of Food Intake (Mini-ECCA) in a Mexican Population
Source: Nutrients. 2018 Apr 23;10(4):524. doi: 10.3390/nu10040524 (PMC5946309; doi:10.3390/nu10040524)
Supplement: Supplementary file 1 [file nutrients-10-00524-s001.zip › Presentation S1 Mini-ECCAΓÇÖs visual aid for food quantity estimation.pdf]

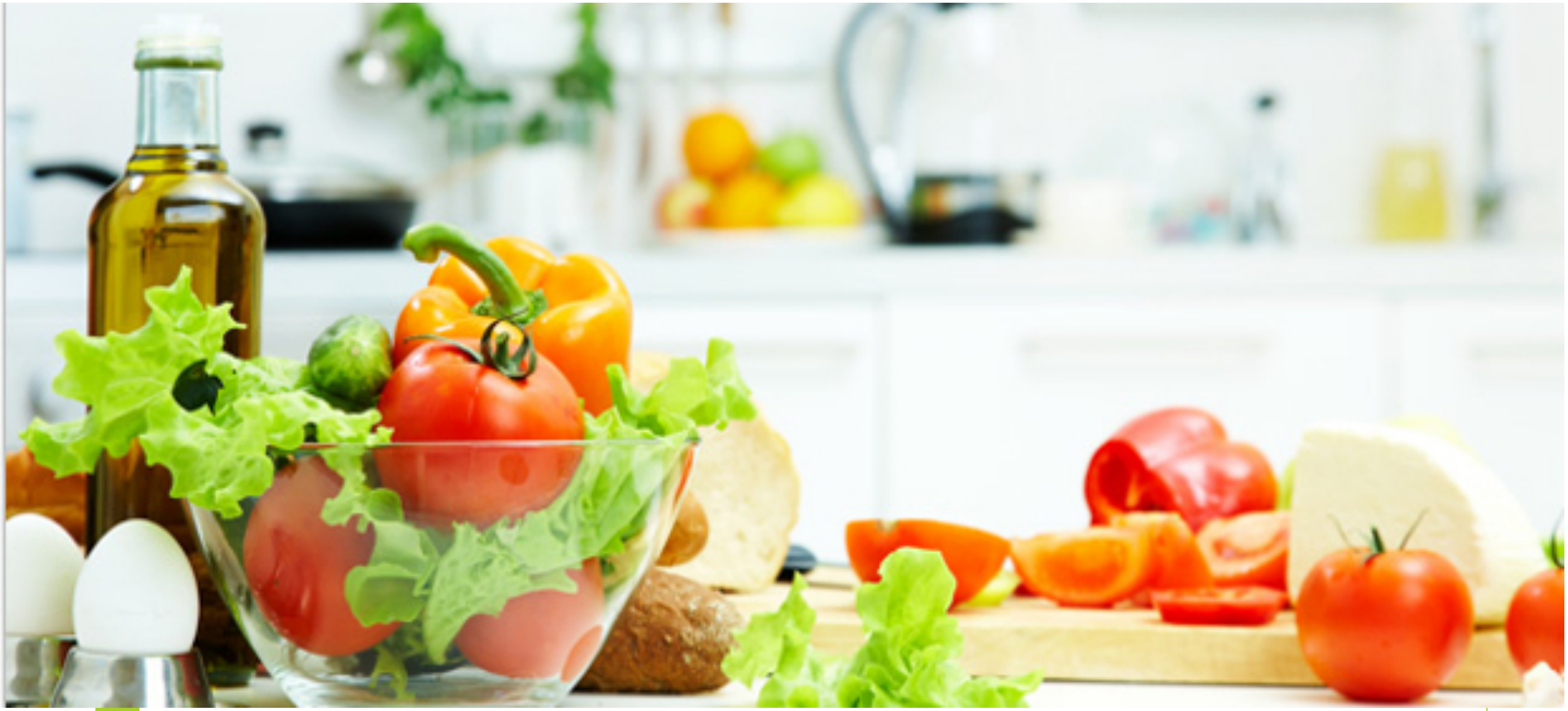

Mini ECCA

Mini Encuesta de Calidad de Consumo Alimentario

1. ¿Bebe, por lo menos, 1.5 litro de **agua natural** al día?

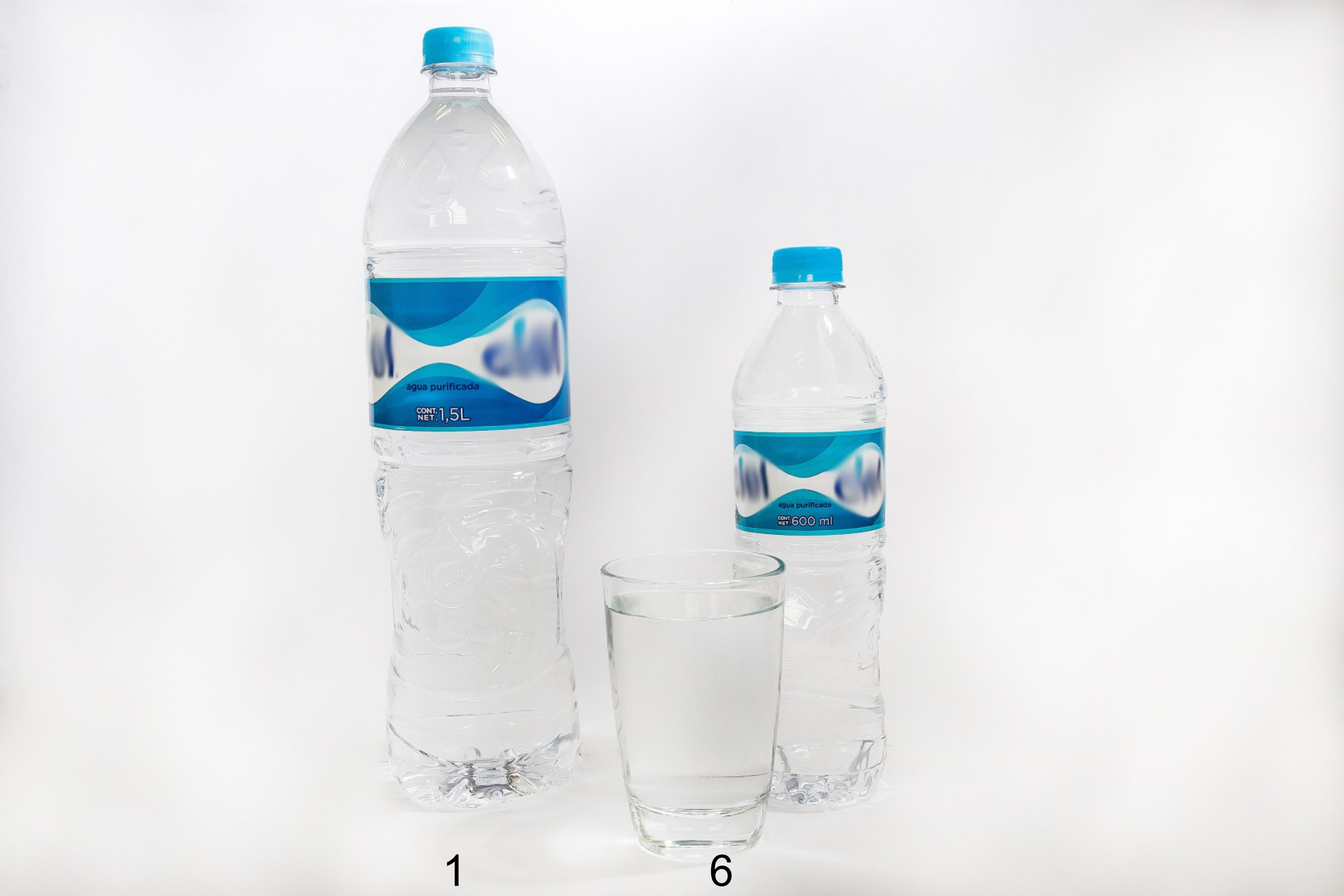

2.5

SI

NO

2. ¿Consume por lo menos, 200g de **verduras** cocidas o crudas al día?

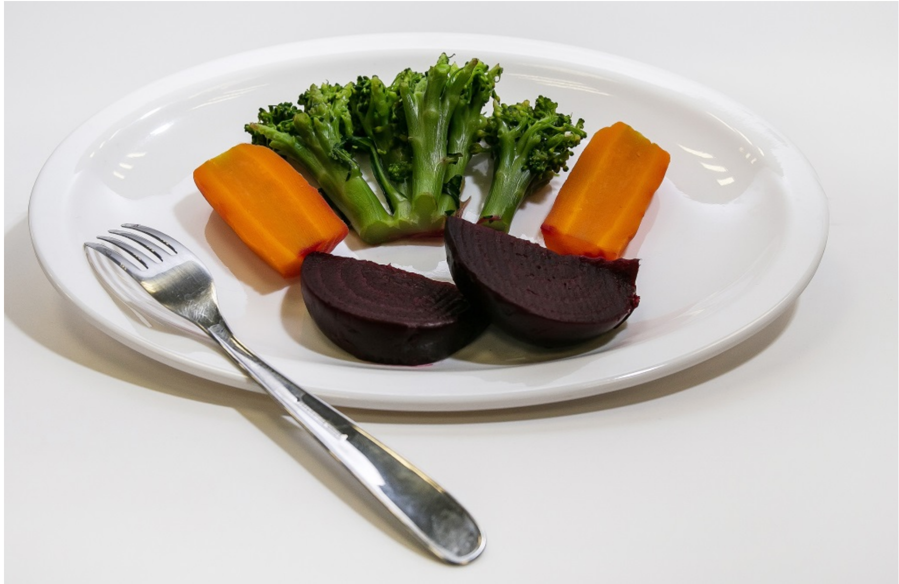

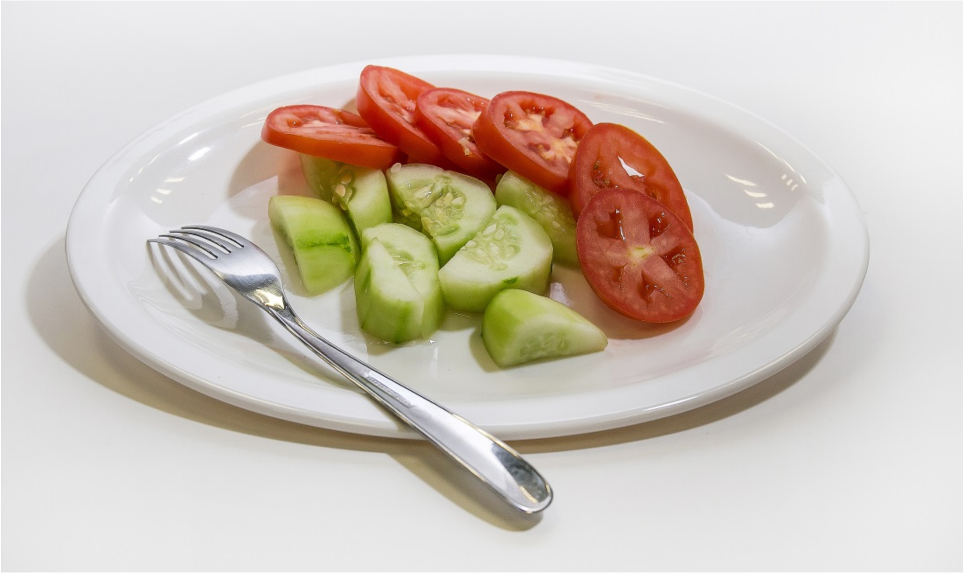

SI

NO

3. ¿Consume **pescado fresco o congelado (100g)** por lo menos, un día por semana?

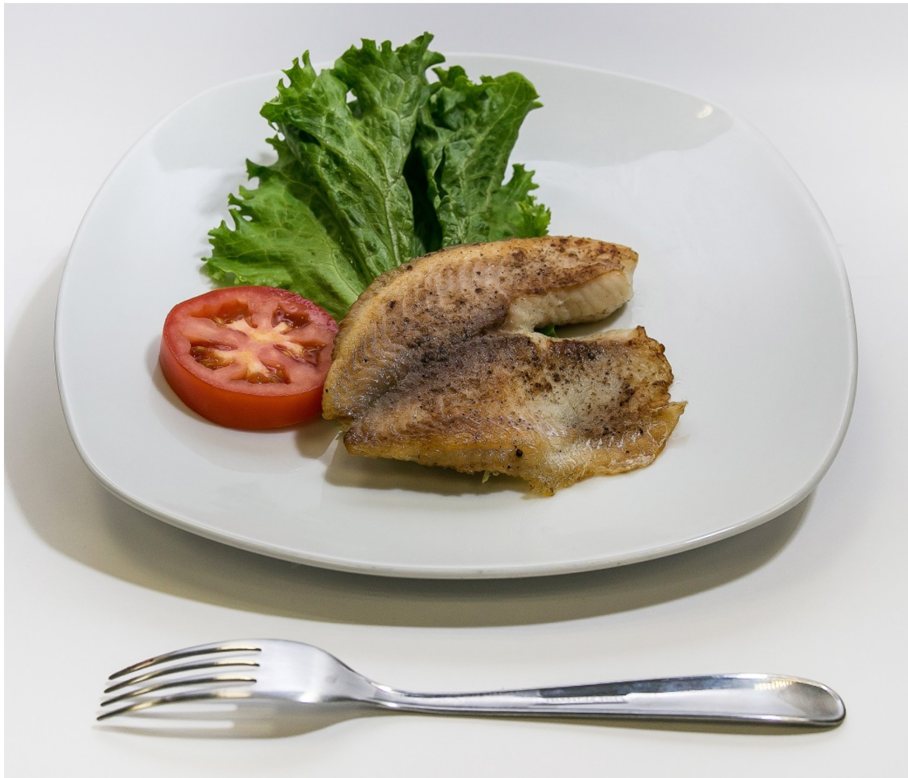

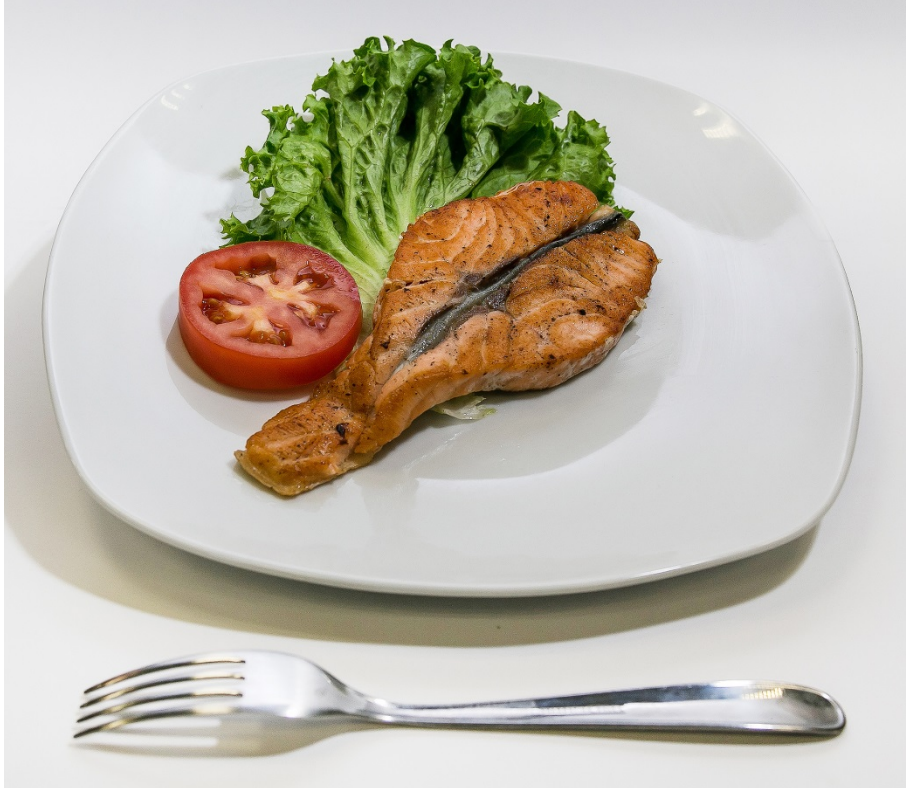

SI

NO

4. ¿Consume 4 ó más **bebidas azucaradas** por semana?

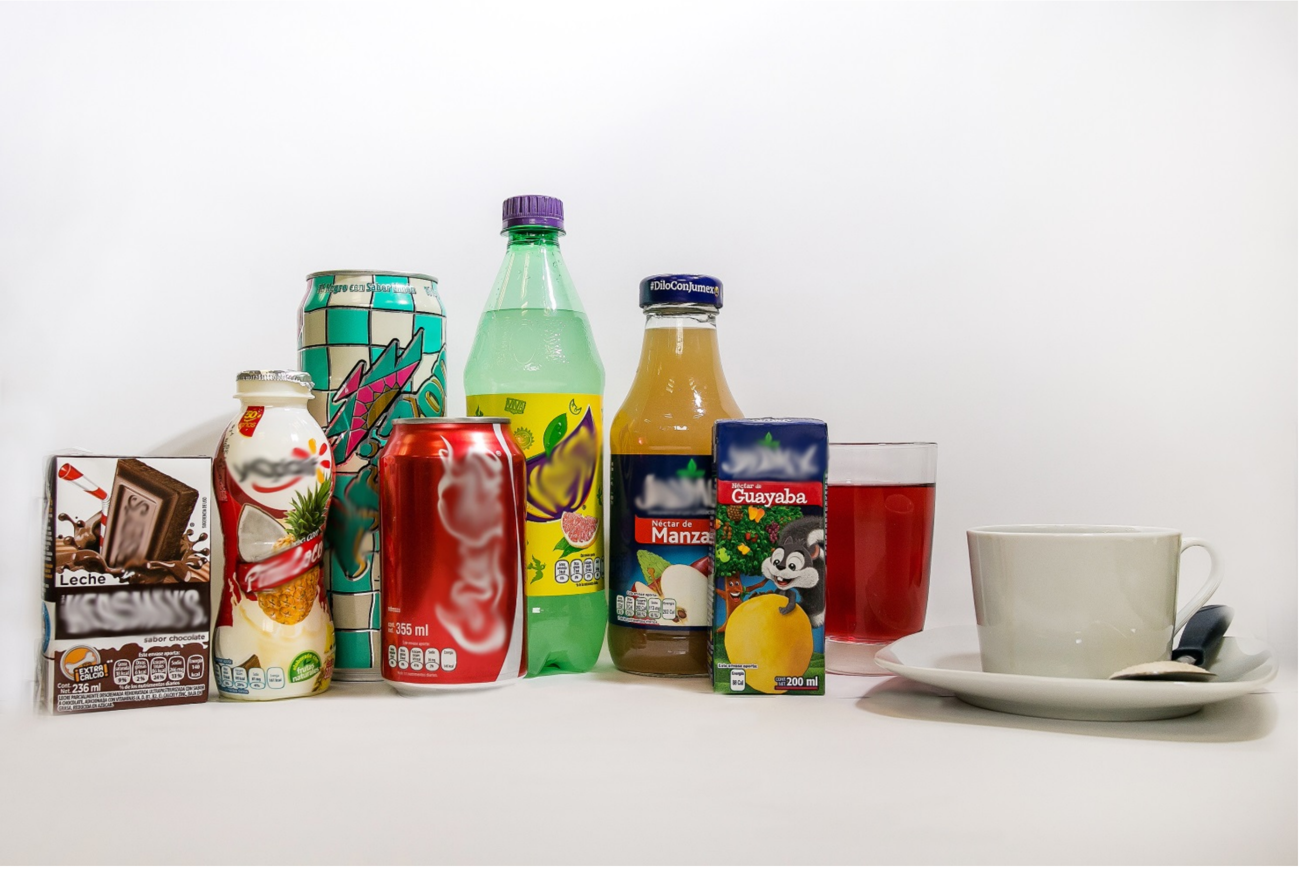

SI

NO

5. ¿Consume por lo menos, 200g de **frutas** al día?

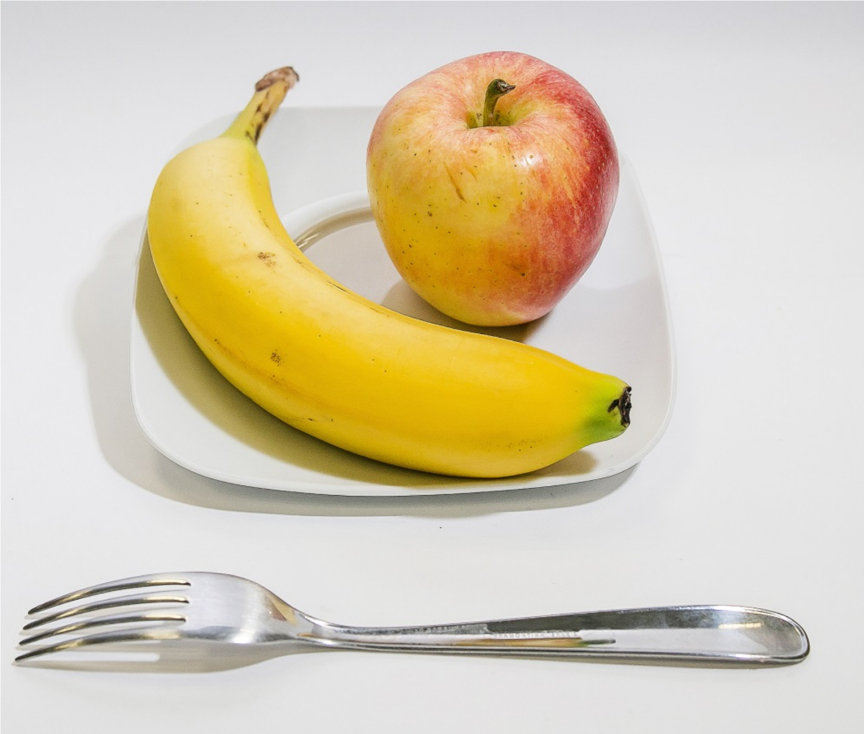

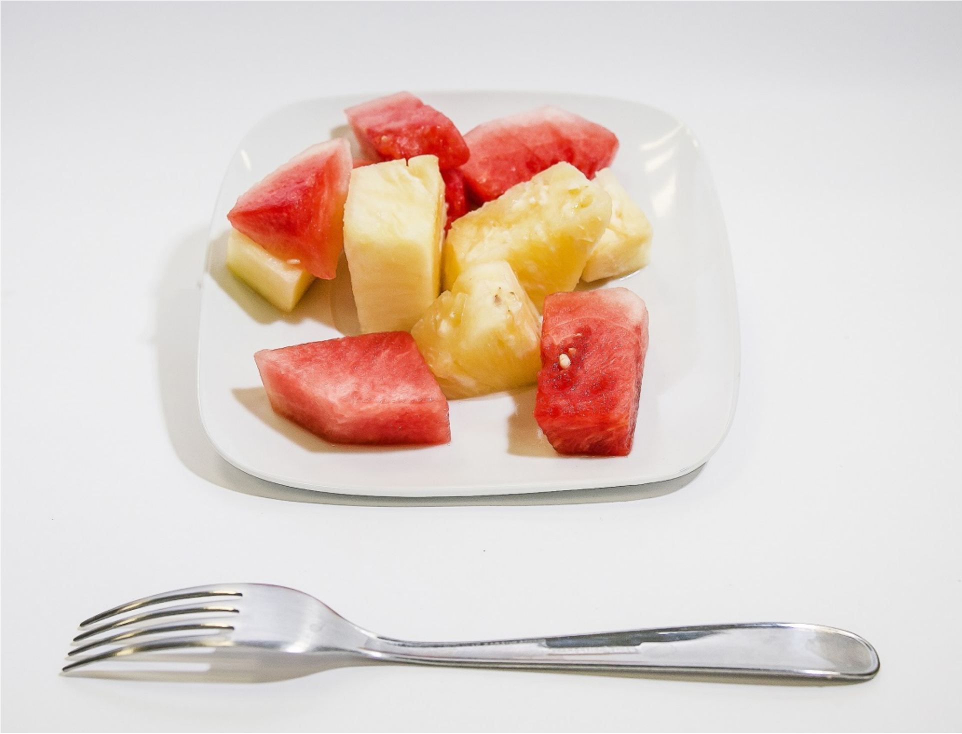

SI

NO

5. ¿Consume por lo menos, 200g de **frutas** al día?

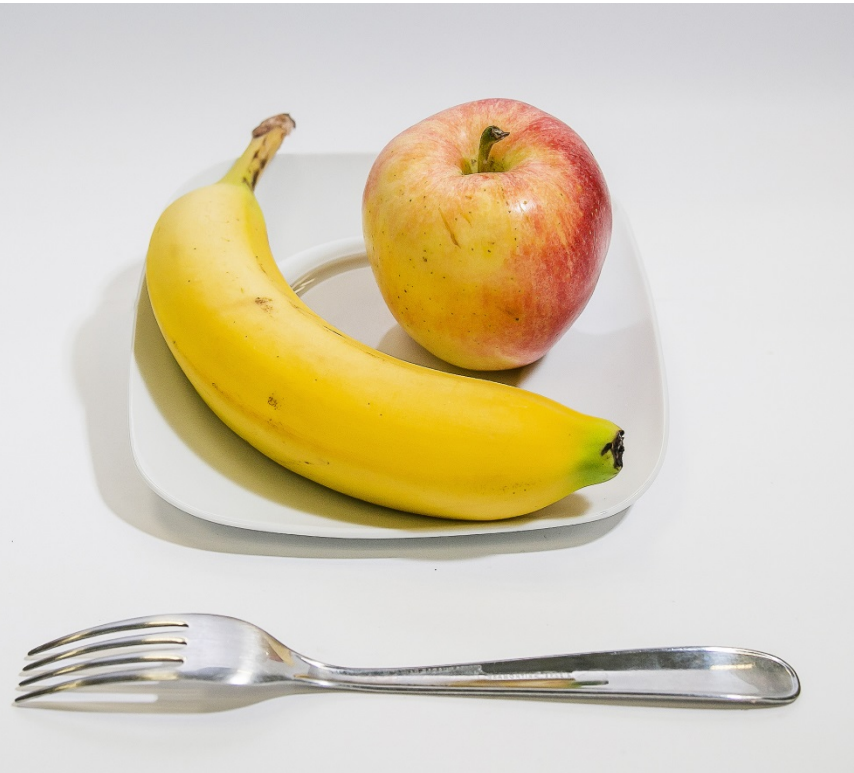

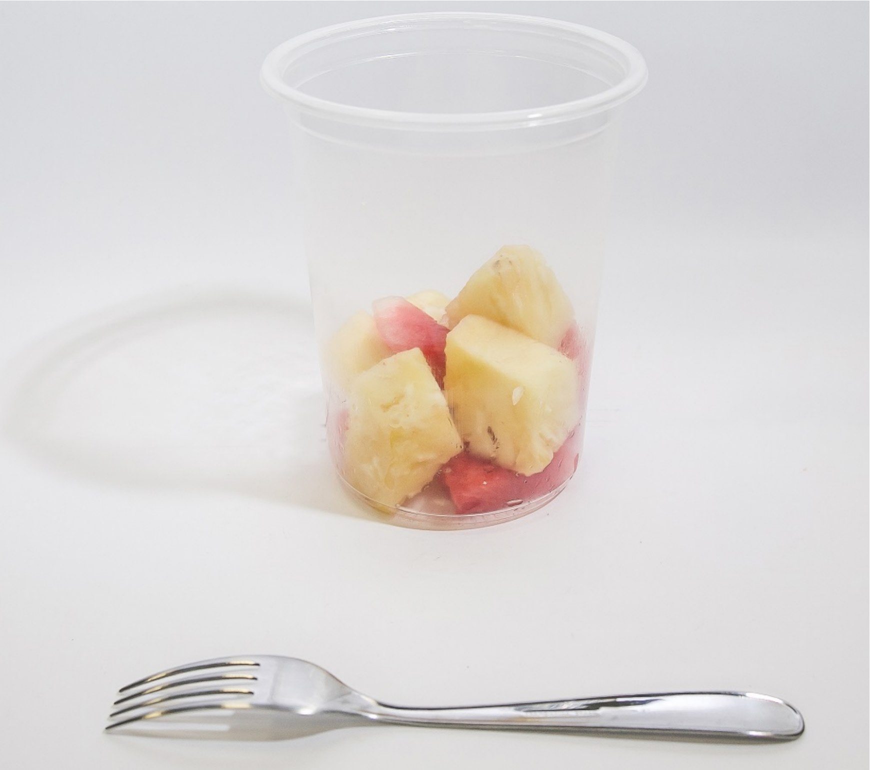

SI

NO

6. ¿Cuál es la **grasa** que consume con mayor frecuencia en la semana?

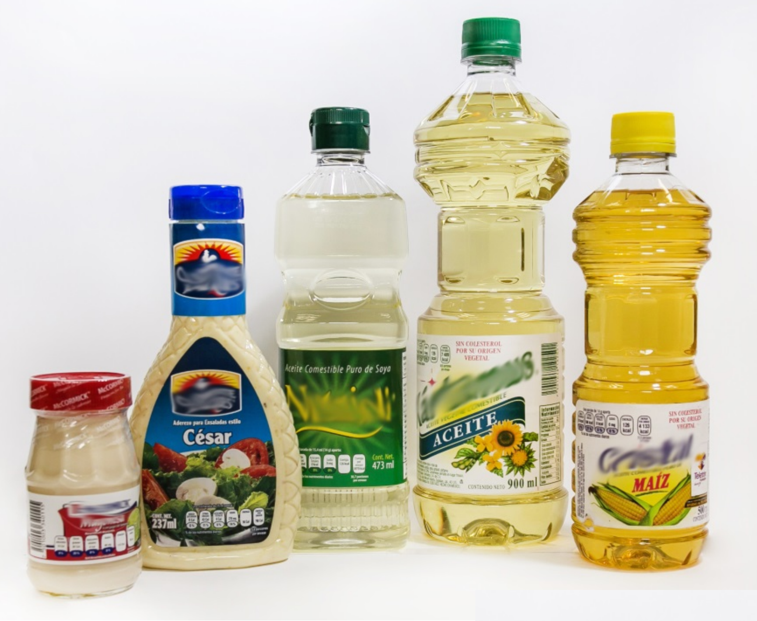

A

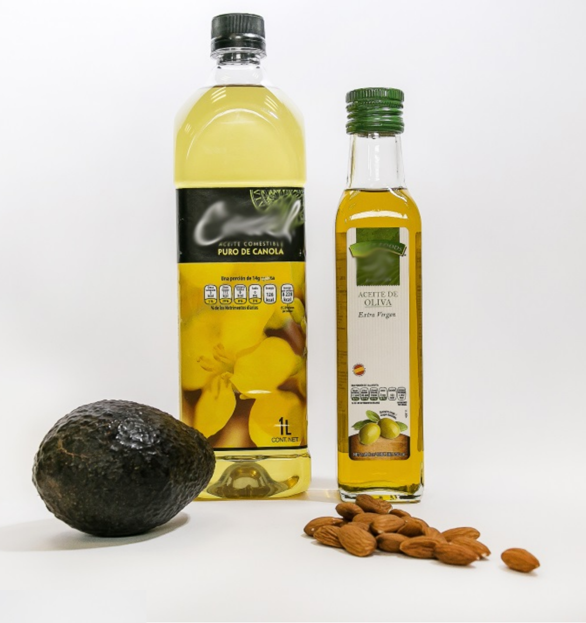

B

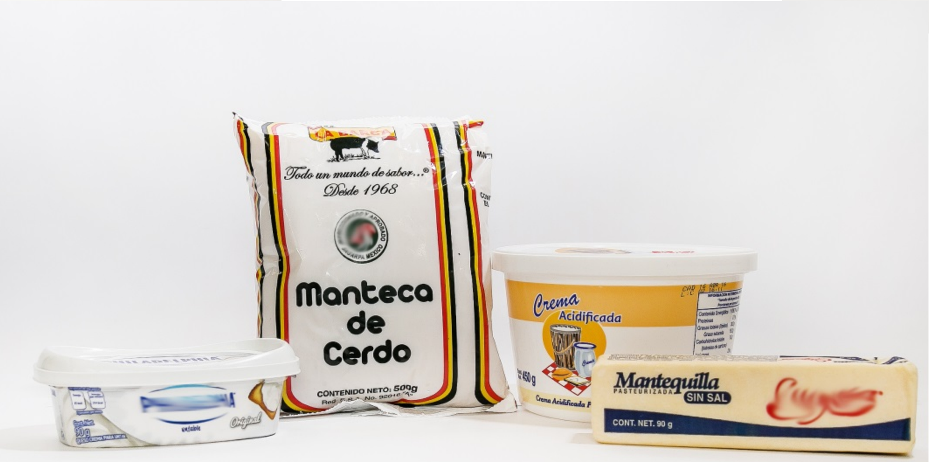

C

No sé

7. ¿Consume **alimentos no preparados** en casa, 3 ó más días por semana?

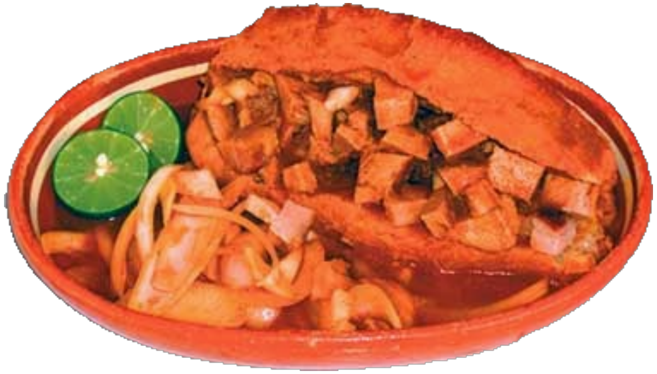

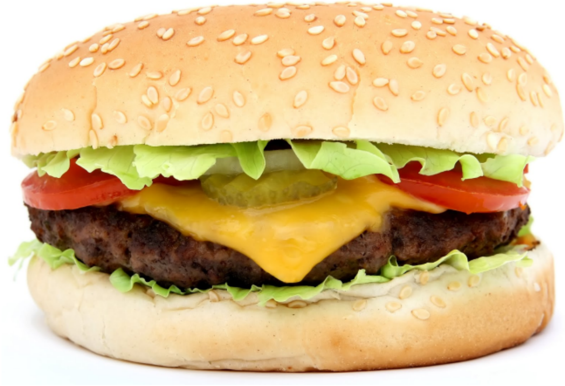

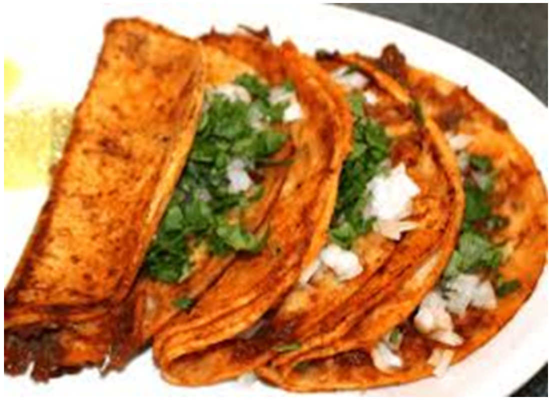

SI

NO

8. ¿Qué tipo de carne consume con mayor frecuencia?

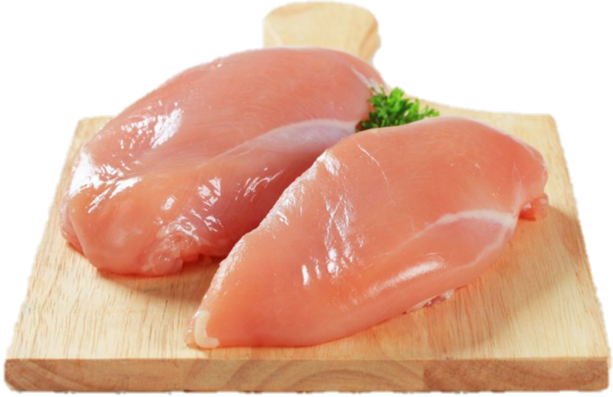

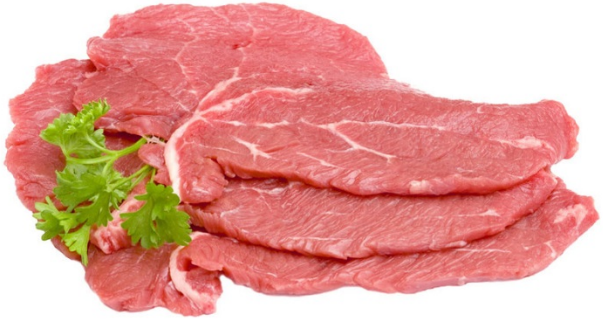

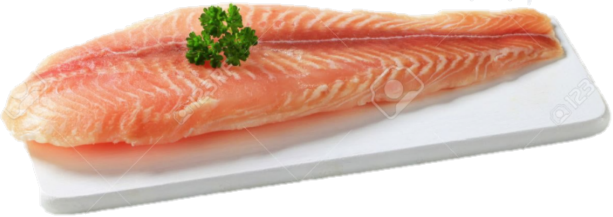

A

B

C

No sé

9. ¿Consume alimentos procesados (frituras, embutidos, platillos empaquetados listos para calentar y servir) 2 ó más días por semana?

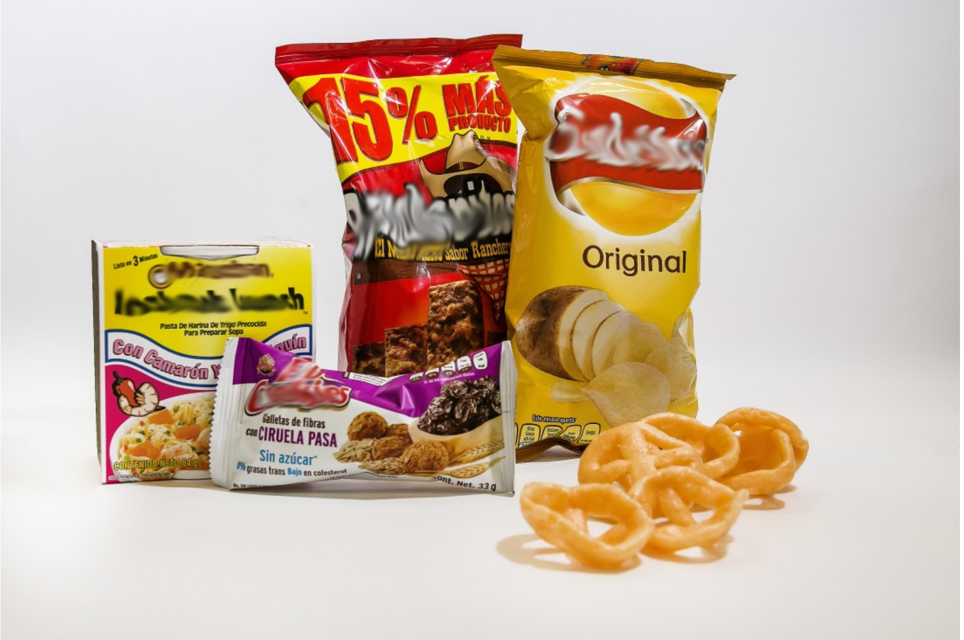

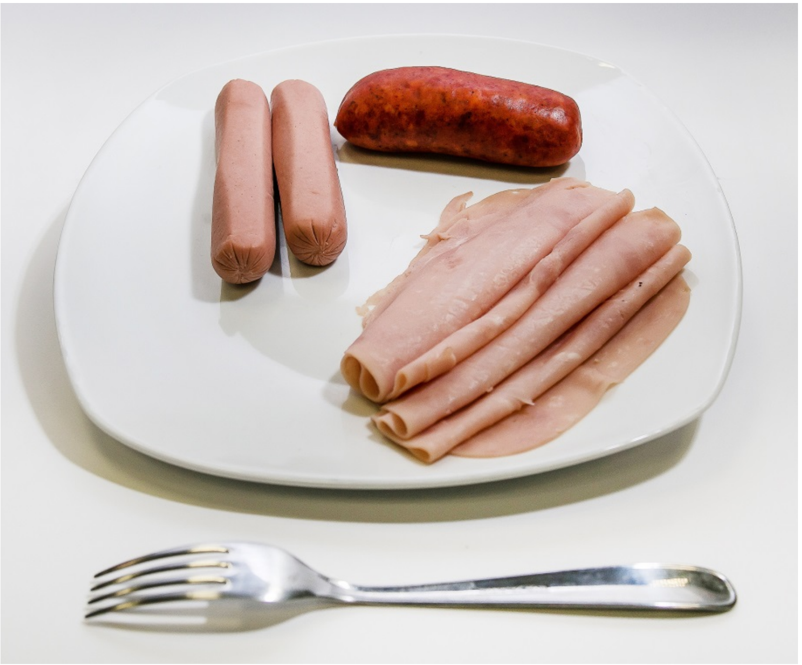

SI

NO

10. ¿Consume **dulces o postres comerciales** 2 ó más días por semana?

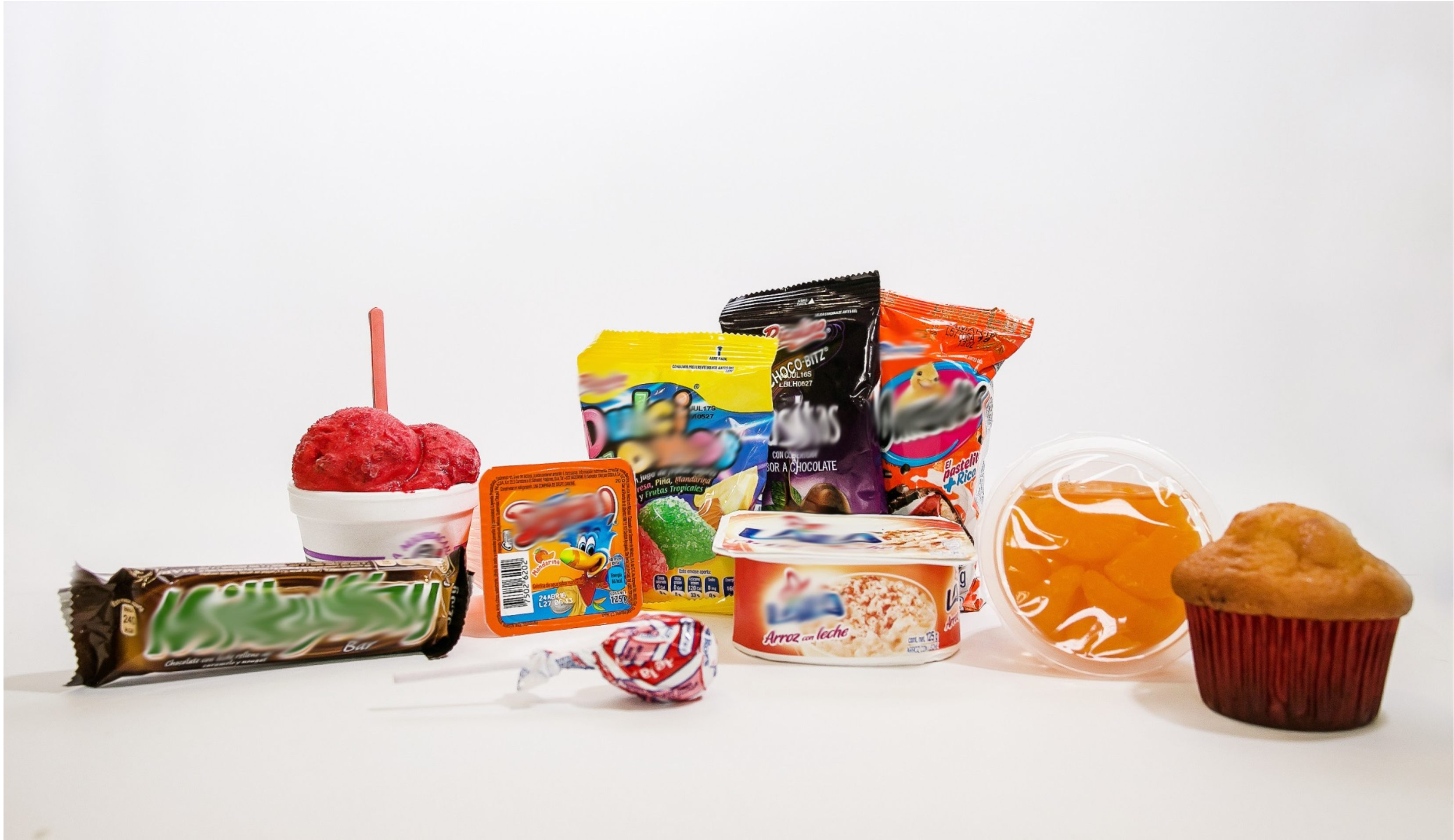

SI

NO

11. ¿Consume **leguminosas** por lo menos, 3 días a la semana (300g a la semana)?

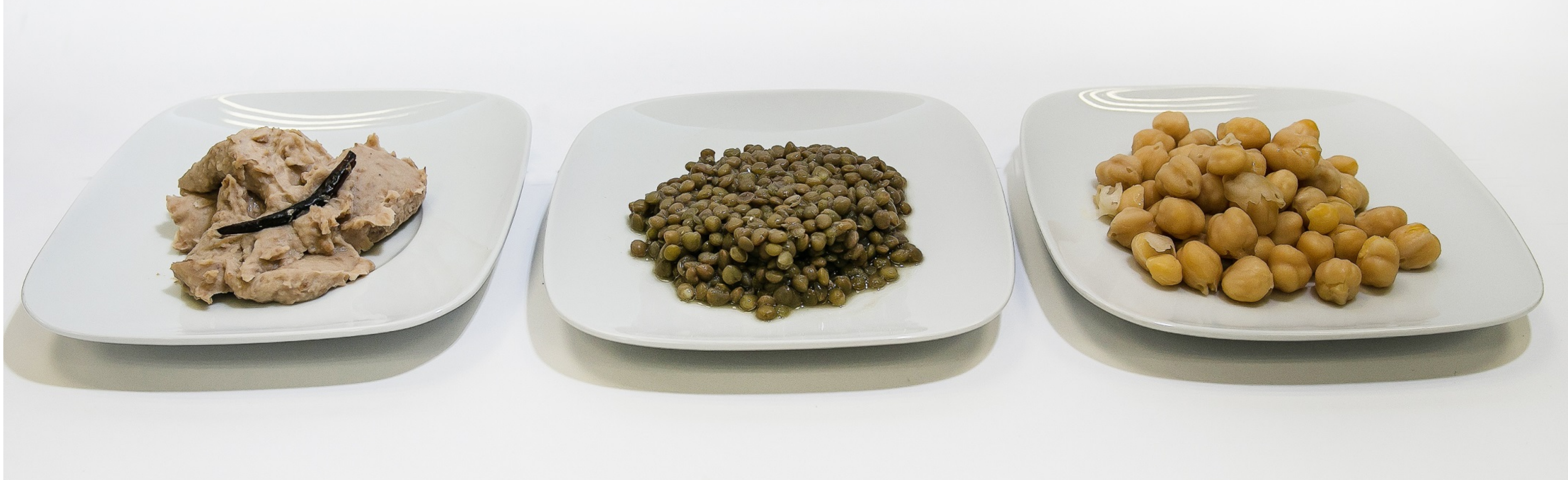

SI NO

12. ¿Qué **cereales** consume con más frecuencia?

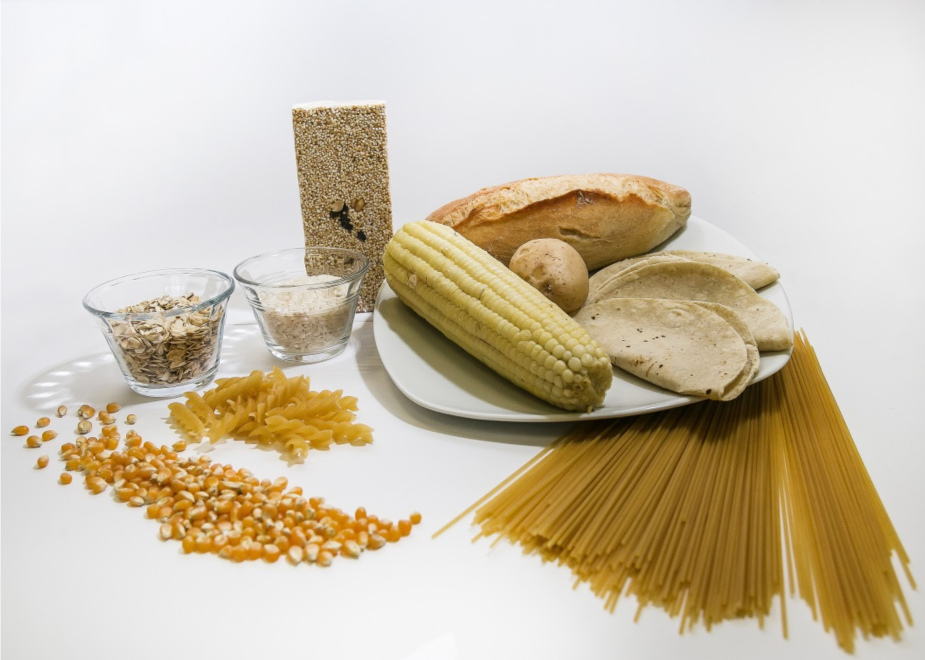

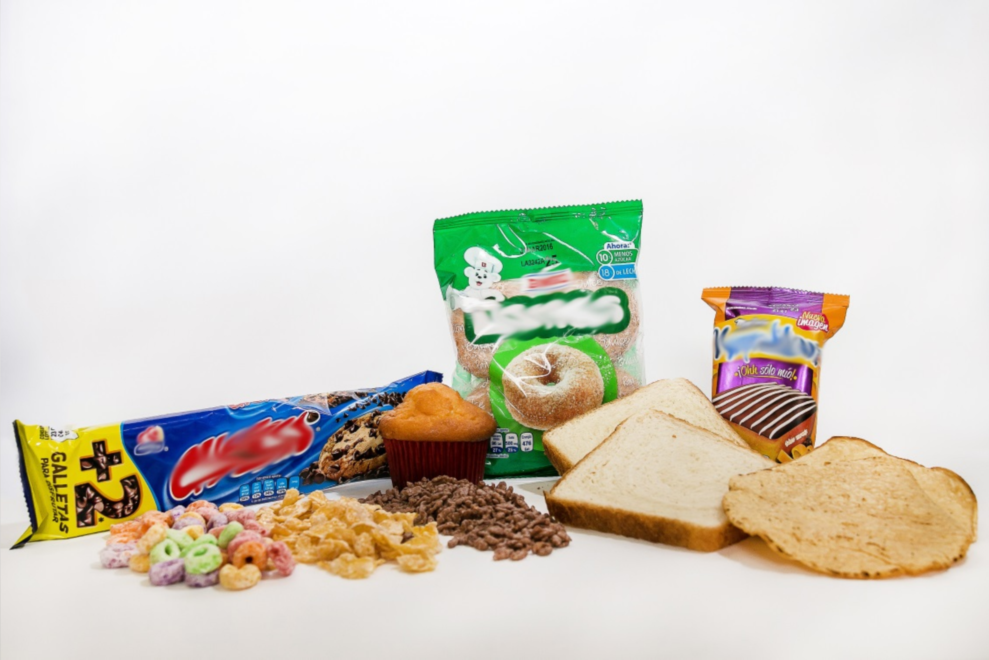

A

No sé

B
