# Supplementary material for: Design and Reproducibility of a Mini-Survey to Evaluate the Quality of Food Intake (Mini-ECCA) in a Mexican Population
Source: Nutrients. 2018 Apr 23;10(4):524. doi: 10.3390/nu10040524 (PMC5946309; doi:10.3390/nu10040524)
Supplement: Supplementary file 1 [file nutrients-10-00524-s001.zip › Table S1 Mini-ECCA survey.pdf]

Table S1: Mini-ECCA survey

No.	Preguntas	Respuestas	Puntaje obtenido
**1**	¿Bebe, por lo menos 1.5 litros de **agua natural** al día?	Sí ☐ No ☐<br>1 0	
**2**	¿Consume, por lo menos, 200g de **verduras** cocidas o crudas al día?	Sí ☐ No ☐<br>1 0	
**3**	¿Consume **pescado fresco o congelado (100g)** por lo menos una vez a la semana?	Sí ☐ No ☐<br>1 0	
**4**	¿Consume 4 o más **bebidas azucaradas** al día?	Sí ☐ No ☐<br>0 1	
**5**	¿Consume, por lo menos, 200g de **frutas** al día?	Sí ☐ No ☐<br>1 0	
6	¿Cuál es la **grasa** que consume con mayor frecuencia en la semana?	A ☐ B ☐ C ☐ No sé ☐<br>0 1 0 0	
7	¿Consume **alimentos no preparados** en casa, 3 o más días por semana?	Sí ☐ No ☐<br>0 1	
8	¿Qué tipo de carne consume con mayor frecuencia?	A ☐ B ☐ C ☐ No sé ☐<br>1 0 1 0	
**9**	¿Consume **alimentos procesados** (frituras, embutidos, platillos empaquetados listos para calentar y servir) dos o más días por semana?	Sí ☐ No ☐<br>0 1	
**10**	¿Consume **dulces o postres comerciales** 2 o más días por semana?	Sí ☐ No ☐<br>0 1	
**11**	¿Consume **leguminosas** por lo menos 3 veces a la semana (300g a la semana)?	Sí ☐ No ☐<br>1 0	
**12**	¿Qué **cereales** consume con más frecuencia?	A ☐ B ☐ No sé ☐<br>1 0 0	

**Cribado:**		
10, 11, 12	Felicitaciones, tu alimentación es saludable	**Total de puntos =**
7, 8, 9	Buen trabajo, estás en el camino correcto.	
4, 5, 6	Algo bajo, podrías mejorar	
0, 1, 2, 3	Estás en la zona de peligro, pero la honestidad es tu valor y te ayudará a mejorar.	
